# Serological Evidence of *Rickettsia* Exposure among Patients with Unknown Fever Origin in Angola, 2016-2017

**DOI:** 10.1155/2020/4905783

**Published:** 2020-08-24

**Authors:** P. F. Barradas, Z. Neto, T. L. Mateus, A. C. Teodoro, L. Duarte, H. Gonçalves, P. Ferreira, F. Gärtner, R. Sousa, I. Amorim

**Affiliations:** ^1^Department of Pathology and Molecular Immunology, Institute of Biomedical Sciences Abel Salazar (ICBAS), University of Porto, Porto, Portugal; ^2^Laboratório De Biologia Molecular, Instituto Nacional De Investigação Em Saúde (INIS), Ministério Da Saúde, Maianga-Luanda, Angola; ^3^CISAS-Center for Research and Development in Agrifood Systems and Sustainability, Instituto Politécnico de Viana Do Castelo, Viana Do Castelo, Portugal; ^4^Escola Superior Agrária, Instituto Politécnico De Viana Do Castelo, Refóios Do Lima, Portugal; ^5^EpiUnit, Instituto De Saúde Pública Da Universidade Do Porto, Porto, Portugal; ^6^Department of Geosciences, Environment and Land Planning Faculty of Sciences, University of Porto, Porto, Portugal; ^7^Earth Sciences Institute (ICT), Faculty of Sciences, University of Porto, Porto, Portugal; ^8^Center for Health Technology and Services Research (CINTESIS), Faculty of Medicine, University of Porto, Porto, Portugal; ^9^Department of Community Medicine, Information and Health Decision Sciences, Faculty of Medicine, University of Porto, Porto, Portugal; ^10^Institute for Research and Innovation in Health (i3S), University of Porto, Porto, Portugal; ^11^Institute of Molecular Pathology and Immunology of the University of Porto (IPATIMUP), Porto, Portugal; ^12^National Institute of Health Dr. Ricardo Jorge, Águas de Moura, Portugal

## Abstract

Spotted fever group *Rickettsia* (SFGR) is one among the aetiologies that cause fever of unknown origin in Angola. Despite their occurrence, there is little information about its magnitude in this country either because it is misdiagnosed or due to the lack of diagnostic resources. For this purpose, eighty-seven selected malaria- and yellow fever-negative serum specimens collected between February 2016 and March 2017 as part of the National Laboratory of Febrile Syndromes, from patients with fever (≥37.5°C) for at least 4 days and of unknown origin, were screened for *Rickettsia* antibodies through an immunofluorescence assay (IFA). Serological results were interpreted according to the 2017 guidelines for the detection of *Rickettsia* spp. Three seroreactive patients had detectable IgM antibodies to *Rickettsia* with an endpoint titre of 32 and IgG antibodies with endpoint titres of 128 and 256. These findings supported a diagnosis of *Rickettsia* exposure amongst these patients and highlight that rickettsioses may be among the cause of unknown febrile syndromes in Angola. Therefore, physicians must be aware of this reality and must include this vector-borne disease as part of aetiologies that should be considered and systematically tested in order to delineate appropriate strategies of diagnostic and control of *Rickettsia* in Angola.

## 1. Introduction

Rickettsioses are vector-borne diseases of medical importance, particularly in African countries where an increasing number of cases have been reported amongst residents and tourists [1]. Despite its public health importance, the epidemiological characteristics linked to rickettsial diseases are poorly defined in the African continent [2]. *Rickettsia* species are strictly intracellular, Gram-negative bacteria from the order Rickettsiales comprising 30 recognized species and numerous uncharacterized strains [3]. Ticks are vectors and reservoirs for several rickettsial agents, but some *Rickettsia* spp. are transmitted by fleas, lice, and mites [4]. These bacteria present several antigenically distinct groups, with those belonging to the spotted fever group (SFG) remaining an important cause of human and animal diseases, characterized by vascular invasion and tissue necrosis [5]. The classical triad of clinical manifestations of SFG *Rickettsia* infection includes fever, eschar, and rash [6]; however, these vary depending on the rickettsial species involved.

In Angola, a high percentage of the population lives in suburban neighbourhoods, characterized by adobe and cement constructed houses with limited access to public basic resources such as potable water, energy supply, health, and education. These highly unhealthy living conditions associated with domestic animals in close proximity increase the exposure to ectoparasites and to the pathogens that they might harbour.

Many studies report rickettsioses acquired by travellers, but the majority refers to sub-Saharan Africa tourists who develop African tick-bite fever (ATBF) [1]. In African countries, fevers of unknown origin can have different aetiologies including rickettsial infection but, due to the overlapping symptomatology with other endemic diseases (e.g., malaria, dengue, HIV, and brucellosis) that also cause fever, as well as the lack of available diagnostic tests and laboratory resources [7], rickettsioses are often underdiagnosed [2].

The aim of this study was to perform the laboratory diagnosis of *Rickettsia* spp. exposure among febrile patients from Angola with malaria and yellow fever already clinically and laboratory discarded.

## 2. Methods

### 2.1. Sample Collection

Between February 2016 and March 2017, a total of 87 serum specimens were obtained from public hospitals, as part of the Febrile Syndrome Surveillance Programme of Angola. These serum specimens were collected from patients from different cities (Benguela, Cabinda, Huambo, Luanda, and Malanje) and provinces (Huíla, Kwanza Sul, Kwanza Norte, Lunda Norte, and Zaire) of Angola and were selected for this study if belonging to individuals presenting fever for at least four days (≥37.5°C) and with at least one of the following inclusion criteria: malaise, myalgia, arthralgia, nausea, vomiting, and rash. These selected serum specimens were also malaria and yellow fever negative, previously tested through peripheral blood smear, malaria antigen detection test (SD BIOLINE), and RT-PCR, respectively.

A questionnaire including patient demographic (age and gender) and epidemiological data (province and municipality of origin, type of residence, household characteristics, season of specimen collection, access to potable water, contact with animals, and clinical manifestations) was filled for each patient by the health care professionals.

### 2.2. Serological Testing

Sera were tested by an in-house immunofluorescence assay (IFA) using *R. africae* strain as antigen, prepared at the Portuguese National Institute of Health Dr. Ricardo Jorge, as previously reported [8]. Along with fever, *Rickettsia* exposure was defined when the sera presented both IgG titre ≥64 and IgM titre ≥32, according to the previously published guidelines for the detection of *Rickettsia* spp. [9].

## 3. Results

**Figure 1:**
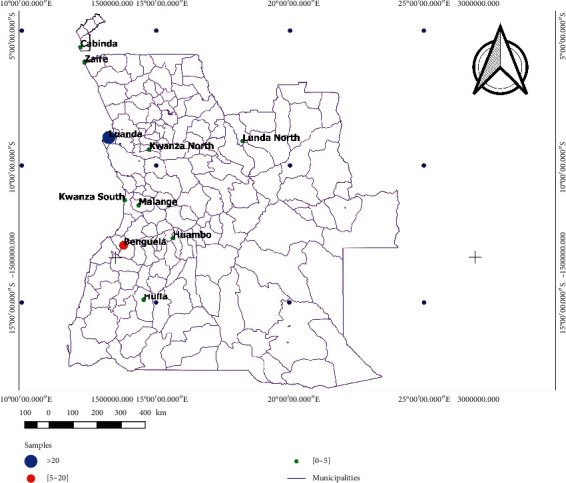
Study area with Angola sampling location cities (Benguela, Cabinda, Huambo, Luanda, and Malanje) and provinces (Huíla, Kwanza Sul, Kwanza Norte, Lunda Norte, and Zaire).

A total of 87 patients from 10 different cities and provinces were analysed in this study (Figure 1). Out of the 87 patients, 27 (31%) were females and 60 (69%) were males. Patients' age ranged between 1 and 86 years, with 45% included in the 13–26 years interval. Eighty-three percent (72/87) of the participants lived in urban areas, while the remaining 17% (15/87) lived in rural areas. All the patients had contact with domestic animals such as dogs, cats, and chickens.

Of all sera from febrile patients of Angola analysed (*n* = 87), three (3.5%; 95% CI: 1.2–9.7) clearly met the laboratory definition of *Rickettsia* exposure. One presented IgG antibody titre of 128 and IgM antibody titre of 32 and the other two seroreactive sera presented IgG antibody titre of 256 and IgM antibody titre of 32. Of the 3 seropositive individuals, two were females and one was a male, with ages ranging from 15 to 34 years, living in Luanda and Benguela cities.

## 4. Discussion

*Rickettsia* spp. is distributed worldwide, but the knowledge about their epidemiology and their health impact in Africa is scarce, with most serological studies focusing on IgG seroprevalence, namely, in South Africa [10, 11], Djibouti [12], Kenya [13, 14], Tunisia [15], Cameroon [16], Zimbabwe [17], Ivory Coast [18], Egypt [19], and Angola [20].

Rickettsioses are rarely considered when evaluating patients with undifferentiated febrile illnesses, and due to the overlapping symptomatology with other endemic diseases such as malaria, dengue, and yellow fever, diagnosis is difficult without confirmatory laboratory tests.

Our study aimed at ascertaining the association of *Rickettsia* exposure with fever of unknown origin by screening febrile patients from Angola that had been previously found to be negative for malaria and yellow fever.

The IFA is currently the gold standard test for serological diagnosis of *Rickettsia* [9, 21]. However, the cross-reactivity of this methodology does not allow the identification of the specific infecting *Rickettsia* species [22]. Several *Rickettsia* antigens should have been tested; however, due to serum sample volume constraints, as well as the availability of IFA slides coated only with *R. africae* antigen, the patient samples were only tested for this SFG species.

This study has detected three *Rickettsia* exposed patients among previously undiagnosed febrile patients (3.5%; 95% CI: 1.2–9.7).

Interestingly, these results are similar to a study reported by Botros and collaborators [19], in which only 1% of the Egyptian garbage collectors tested presented seroreactivity against *R. conorii*. Nevertheless, the herein study presents lower seroreactive serum samples when compared with other reports, demonstrating 17.68% of human *Rickettsia* exposure in Reunion Island, Southern Africa [23]; 21% in febrile patients from Mpumalanga, South Africa [10]; 24.1% in a pastoral HIV-endemic community of South Africa [11]; 16% in workers from a Djiboutian abattoir in East Africa [12]; 10% of febrile patients from Kenya; 22.4% of febrile children from western Kenya [14]; 66% of patients with fever of undetermined origin from Tunisia [15]; 32% of febrile patients from Cameroon [16]; and finally, 5.3% and 6.2% of a rural population of Sierra Leone and Ivory Coast, respectively [18].

Despite the important message that our results may arouse regarding a possible *Rickettsia* exposure, for several reasons, they should be carefully analysed and interpreted. According to Brouqui and collaborators [24], *Rickettsia* IgM and IgG antibodies are usually detected seven to 15 days after disease onset. The patients herein tested presented fever for at least 4 days. However, we cannot know exactly how long this clinical manifestation lasts, which makes it impossible to critically contextualize with the respective serological data. On the contrary, it is important to be aware that IgM cross reactions with other pathogenic agents or false-positive IgM antibodies observed, for instance, when rheumatoid factor is present, may occur, as described in the guidelines for the detection of *Rickettsia* spp. [9]. Even though, our detected cases of IgM positive specimens were accompanied with positive titres of IgG (128 and 256) which, taken together, may reinforce the premise that rickettsiae are circulating in Angola. Nevertheless, and ideally, in order to confirm a current rickettsiae infection, these patients should be retested and checked for seroconversion or increased antibody titres in matched samples at 3-week intervals.

The transmission and dissemination of rickettsiae through vectors are a phenomenon of growing concern with the expanding human populations and increasing contact between humans and animals (domestic and wildlife) [25].

The rickettsiae-exposed patients who participated in our study lived in urban zones of Benguela and Luanda cities. One of them is a student, and the other two street vendors. A previous study done in pet dogs from Luanda [26] described a low *Rickettsia* seroprevalence in these animal species. Probably, ticks and fleas competent for *Rickettsia* appear with a low rate of parasitism in Angola.

Although in a low prevalence, this finding is relevant to the clinical management of patients with fever of unknown origin and support the inclusion of this VBD in clinical diagnostic algorithms.

To conclude, our findings suggest that rickettsioses are present in Angola and, therefore, should be taken into account in cases of febrile illness. The serological evidence of exposure with these bacteria raises attention for the need of appropriated public health interventions and diagnostic improvement. Forthcoming studies should include a higher specimen number, with the possibility of detecting the pathogen in acute infection phases, both acute and convalescent samples screening, antibodies testing against several antigens, and, if possible, application of molecular techniques in skin biopsy or swab samples from suspected cases with eschar. This will allow the identification of risk factors and the establishment of prevention and control disease strategies for *Rickettsia* spp. infection.

## Data Availability

Data used to support the findings of this study are available from the corresponding author upon request.

## Abbreviations

ATBF: African tick-bite fever
IFA: Immunofluorescence assay
Ig: Immunoglobulin
SFGR: Spotted fever group *Rickettsia*
RTPCR: Reverse transcription polymerase chain reaction.
